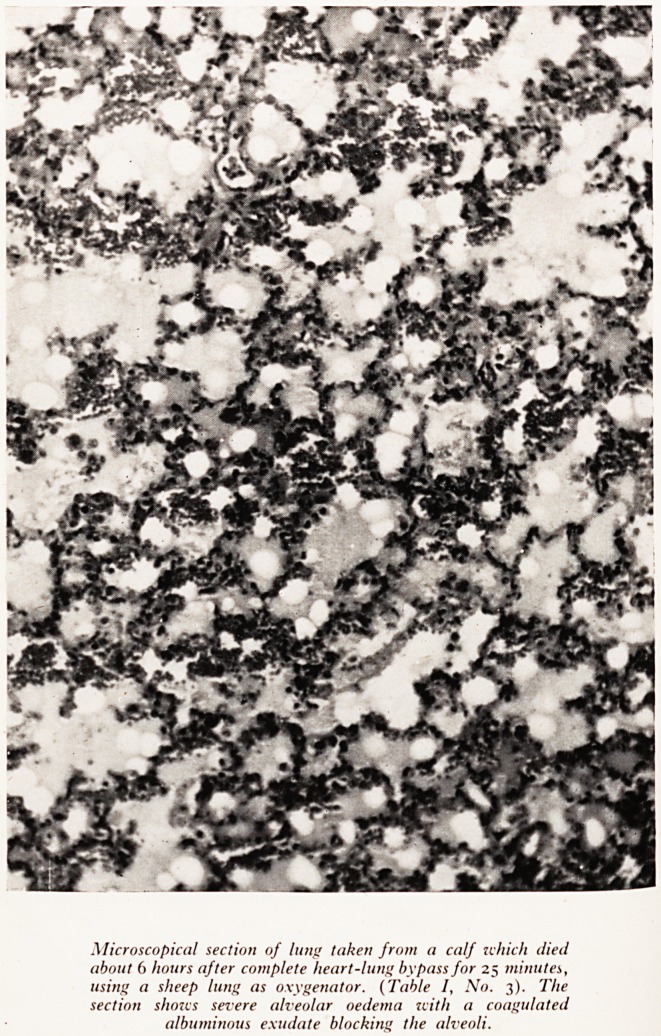# The Use of an Animal Lung as Oxygenator in a Heart-Lung Machine

**Published:** 1959-10

**Authors:** M. G. Wilson, R. E. Horton, B. G. Barratt-Boyes, J. Clutton-Brock

**Affiliations:** From the Departments of Surgery and Anaesthetics, UNIVERSITY OF BRISTOL; From the Departments of Surgery and Anaesthetics, UNIVERSITY OF BRISTOL; From the Departments of Surgery and Anaesthetics, UNIVERSITY OF BRISTOL; From the Departments of Surgery and Anaesthetics, UNIVERSITY OF BRISTOL


### THE USE OF AN ANIMAL LUNG AS OXYGENATOR IN A HEART-LUNG MACHINE

BY

M. G. WILSON, CH.M., F.R.C.S.,
R. E. HORTON, M.B.E., M.S., F.R.C.S.
B. G. BARRATT-BOYES, M.B., F.R.A.C.S.
J. CLUTTON-BROCK, M.A., M.B., B.Chir., F.F.A.R.C.S.

*From the Departments of Surgery and Anaesthetics,*
*UNIVERSITY OF BRISTOL*

Much of the progress in modern cardiac surgery has been made possible by inventions which mechanically oxygenate blood outside the body and pump this blood back into the circulation—"the heart and lung bypass."

In 1955, when our work was first planned, the only successful heart and lung bypass reported in man was that of Gibbon (1953). A variety of other mechanical oxygenators were being developed, notably those of Melrose (1953), Bjork (1948), Denis (1951), Helmsworth (1952), Dodrill (1952), Jongbloed (1949), using either filming or bubbling as the method of oxygenation but success with these was, at that time, elusive and it seemed possible that there was some inherent defect in the direct exposure of blood to gas. There were also the obvious dangers of gas or fibrin emboli, the difficulty of cleaning large oxygenating surfaces and the bogey of the denaturing of proteins. Mustard (1952) suggested that the lung might also perform some essential function other than gaseous exchange. It seemed therefore that this method was worthy of further trial, and this paper records our experiences in using the lungs of one animal to oxygenate blood from another animal undergoing bypass operations.

*Choice of species for animal experiments.*—In most other laboratories the experimental animal for this type of work has been the dog. It seemed to us, however, that ruminants—calf, goat and sheep might have advantages over the dog. Among these advantages were larger vessels for cannulation, and a blood flow approaching that of man. With calf and sheep unlimited amounts of blood could be obtained from the abbatoir for priming the machine and for transfusion.

With animals of differing species to work with, it would be possible to test out heterologous lungs. Finally, if the method could be applied to man a lung about the same size as man's would obviously be an advantage.

### METHODS

*Preparation of the isolated donor lung* was based on the technique described by Campbell (1956). The lungs were obtained on the day of the experiment, the technique varying however, depending on whether the goat or the sheep was the donor animal. (*a*) Goat lungs. These were obtained in the experimental laboratory using aseptic technique. To prevent clotting of blood in the lungs the goat was heparinized (150 mgm.) and anaesthetised with thiopentone. To avoid collapse of the lungs on opening the thorax and also inhalation of ruminal content, the trachea was exposed in the neck and clamped. The thorax was then opened and a clamp placed across the main pulmonary artery. The trachea was clamped high in the thorax and divided above the clamp. The heart and fully expanded lungs were removed from the chest by dividing the aorta, great veins and fibrous attachments. The heart, with the exception of a cuff of right ventricle around the pulmonary valve and the portion of left atrium receiving the pulmonary veins was cut away. The lungs were weighed. The main pulmonary artery was cannulated through the intact pulmonary valve using a perspex cannula, and the clamp removed. By clamping the pulmonary artery before dividing any of the great veins and by having the perfusion fluid running continuously through the cannula, air embolism to the lung was prevented. In the earlier experiments the main pulmonary artery was cannulated directly: this proved less satisfactory, some air occasionally entering the artery. A second cannula was tied into the trachea and connected to a specially designed mechanical respirator (Clutton Brock 1957) which inflated the lungs intermittently with 100 per cent. oxygen at a pressure from 0–20 cm. $H_2O$, using a sine wave pattern. The lungs were then continuously perfused with dextran solution using the venous pump of the machine at a low flow rate (200–400 ccs. per min.). A depulsator was placed in the circuit proximal to the pulmonary artery to lower the pulse pressure. The mean pulmonary artery pressure was not permitted to exceed 20 mm.Hg. Five pints of dextran were recirculated through the lungs for 10–15 mins., then replaced by a further 5 pints and so on until the dextran remained clear. A total of 15–20 pints was required for a lung weighing 800–1,000 gms. Great care was taken to avoid air embolism during the washing and during the periods when recirculation was stopped ventilation of the lungs was also discontinued, because it was possible that ventilation without perfusion may result in air being sucked into the lungs from the open pulmonary veins (Campbell 1956—personal communication). In some earlier experiments a simple non-pulsatile gravity wash was tried. While this method provided effective washing it required a greater quantity of dextran. Gum saline solution was also tried as the perfusion fluid, but several lungs became severely oedematous during washing, and analysis showed the pH of the gum saline to be variable. When washing was judged complete a sample of the final effluent was taken for analysis of red cell content, and the machine and lung were primed with blood. The first 200 cc. of blood pumped from the lungs contained dextran and was discarded.

(*b*) Sheep lungs: These were obtained from sheep killed at the city abattoir. The animal was temporarily stunned by an electric shock and in approximately half the cases heparin was injected intravenously—to keep the animal subdued several shocks were necessary. The throat was cut and the animal bled to death. Before the butcher opened the thorax (about 10–15 minutes later) the trachea was clamped at the neck. After opening the thorax the technique was identical to that in the goat—the heart and lungs being removed by one of us. It was frequently found that the lungs were not fully inflated and, in later cases, the trachea was immediately cannulated, the lungs blown up by mouth and the tracheal clamp replaced. When this was not done the lungs were difficult to ventilate during washing. The heart and lungs were placed in a plastic bag and transported to the experimental laboratory. There was thus an unavoidable delay of an hour or more before washing was commenced.

*Apparatus:* The machine we used is shown in Fig. 1.

The pumping and control apparatus was that designed by Melrose at the Post-graduate Medical School, Hammersmith (Cleland and Melrose, 1955). A sloping container (S) held one or two pairs of lungs (L) and blood from the lungs drained into a calibrated perspex arterial reservoir (AR). Blood passed through the lungs, out of the open pulmonary veins and filmed down the slope into the arterial reservoir in an unbroken stream without frothing. A 100 mesh Monel wire filter was placed over the outlet from the arterial reservoir. The circuit contained in addition a priming reservoir (PR), a venous reservoir (VR) and adextran reservoir of siliconized glass (not shown). The depulsator (D) was made of a length of thin latex "Paul's tubing" two or three ply stretched between perspex fittings. An arterial and venous bubble trpa (ABT, VBT) were placed in the circuit distal to the arterial and venous pumps and pulmonary artery pressure was continuously monitored by an electromanometer *via* a side arm in the pulmonary artery line. The tubing was poly-vinyl chloride (3/8 ins. int.-diam.) and all extra connectors were of perspex designed to provide a continuous smooth lining and prevent turbulence.

FIG. 1. *Diagram of Heart-Lung machine circuit. For description see text.*

VL = Venous line. VR = Venous reservoir. VP = Venous pump. D = Depulsator. VBT = Venous bubble trap. L = Lung. S = Sloping container. AR = Arterial Reservoir. AP = Arterial Pump. ABT = Arterial Bubble Trap. AL = Arterial line. CSL = Coronary sinus line. CSP = Coronary sinus pump. PR = Priming reservoir. MR = Mechanical respirator.

The apparatus was sterilized by standing for twelve hours in 1:10,000 benzalkonium chloride solution (Roccal). This was washed out first with tap water, then sterile saline. The venous pump was then used to wash the lungs with dextran *via* an additional connection between the arterial reservoir and the venous line. The machine and lungs were then primed with blood obtained on the morning of the experiment, 25 mgm. of heparin in 50 ccs. 5 per cent. dextrose solution being added to each pint.

During by pass the amount of blood contained in the isolated lung varied according to the rate of flow. In order to keep the amount of blood in the apparatus constant at different rates of flow during bypass the arterial reservoir was calibrated by pumping blood through the lungs at different rates of flow and noting the level of blood in the arterial reservoir. There was a difference of 200 to 350 ml. between the resting level and the level at a flow rate of 2 litres per minute. During perfusion the level of blood in the reservoir was kept at the level appropriate for the rate.

### OPERATIVE TECHNIQUE USED IN ANIMAL EXPERIMENTS

In most experiments a calf from 1 to 8 weeks old (average weight 40 kg.) has been used as the subject for bypass operation, and lungs from a goat or sheep have been used in the machine. In a few early experiments a goat was used as the subject with goat lungs in the machine and in one case a sheep was used with goat lungs in the machine.

Premedication was with atrophine, and promethazine (phenergan). Anaesthesia was induced with thianbutone (ulbreval) and maintained, in the earlier experiments, with nitrous oxide and oxygen and small doses of thianbutone and later experiments with nitrous oxide and oxygen and a small amount of trilene. Also in later experiments an electric blanket was placed underneath the animal to counteract the fall in body temperature which occurred during perfusion. E.C.G. and E.E.G. records were taken before, during and after bypass operation.

The incision was in most cases a right thoracotomy through the fifth interspace with division of the 2, 3 and 4 costal cartilages. When this was completed heparin, 3 mgm/Kg, was given and the femoral artery and vein cannulated with fine polythene tubing for measurements of pressure in the abdominal aorta (by Hg. manometer) and inferior vena cava (by saline manometer). The right subclavian or carotid artery was then exposed and a cannula inserted into it and directed centrally. The superior and inferior venae cavae were then taped and cannulated through the right atrial appendage. The venous blood was drained by gravity into the venous reservoir placed six to twelve inches below the level of the right auricle. In the earlier experiments blood was aspirated direct from the cavae by the venous pump: following introduction of the gravity method a smoother higher flow was maintained and by varying the height of the venous reservoir the level of central venous pressure was easier to control, the rate of flow being adjusted to maintain a normal central venous pressure. Throughout the bypass the subject's lung was kept inflated, in the early experiments by clamping the intratracheal tube, and later by continuing rhythmic ventilation with nitrous oxide and oxygen. As the object of the experiment was to test the isolated lung oxygenator, a cardiotomy was done in only a few cases. Total heart-lung by pass was maintained in most cases for 30 minutes. The caval tapes were then released and pump rates gradually diminished. A fall of blood pressure occasionally necessitated a return to partial bypass and administration of nor-adrenaline. Great care was taken to maintain an accurate blood balance by equating operative blood loss with addition of blood to the circuit. After the cannulae were removed, 200 mgs. protamine sulphate was usually found to be sufficient to return the clotting time to normal. Both thoracic cavities were connected to underwater drainage for the first four to eight postoperative hours. Samples of blood were taken for electrolyte, pH, plasma Hb, cell counts and oxygen estimations. The saturation was measured by the pipette method of Roughton and Schollander (1943) or by Handforth's method (1959) using an EEL colorimeter. Specimens were taken from the donor lungs after perfusion and from the lungs of the animals failing to survive.

### RESULTS

An attempt was made to by-pass the heart and lungs in forty experiments.

In twelve experiments performed to gain technical experience a goat was used as the subject and goat lungs in the oxygenator. All these animals died. The lungs were not heterologous and the technique was elementary, so the results will not be given in detail; suffice it to say that in all cases the lung oxygenated satisfactorily and that in most cases death was due to obvious technical errors.

There were twenty-eight experiments in which an adequate flow was maintained with a heterologous lung for a sufficient time to assess its behaviour as an oxygenator. In all but one a calf was the operated subject. These experiments fall into two groups:

Group I—Oxygenator lungs taken from sheep in abattoir three hours or more before use.

Group II—Oxygenator lungs taken from goat in laboratory one and a half hours or less before use.

*Group I. Heterologous Sheep Lungs. See Table I.* In seven of the ten experiments, two pairs of lungs were used for the perfusion, connected in parallel. The delay before using these lungs was between two and three hours and ventilation of the lungs was often difficult, higher pressures being required than in the other groups. Dextran was used for washing in all cases. In the majority the wash, as judged by the red cell count of the final effluent, was not complete. In five experiments (Nos. 1–5) the sheep was not heparinized and in at least two of these some clotted blood was noted in the wash from the lung. Oxygenation was usually satisfactory although in three of the eight experiments where two pairs of lungs were used one pair was noted to be oxygenating poorly and was therefore excluded from the circuit. In Exp. No. 10 the single lung failed to oxygenate adequately after 10 minutes perfusion and developed gross oedema.

The development of pulmonary oedema in the oxygenator lung was less severe when the donor was heparinized: in these lungs the percentage weight increase was 42 per cent. while in the unheparinized it was 51 per cent. One animal in this group survived despite the development of gross oedema in the donor lung.

*Group II. Heterologous Goat Lungs. See Table II.* In all except No. 14 the goat was heparinised. By correctly timing the killing of the goat the lungs were used for the by pass in less than 1½ hours. The completeness of the wash was good in all cases, the red cell count in the final effluent falling as low as 3000 cells/c.mm. A cell count of 5000/c.mm. represented a dilution of the blood in the lung by 1/1000; if there were 200 c.c. of blood in the lungs before washing only 0·2 ccs. would remain to be mixed with the circulation of the subject. Oedema was insignificant or absent in thirteen, moderate or severe in five. The development of oedema seemed to bear no relationship to the duration of the perfusion or to the pulmonary artery pressure. Oxygenation was adequate even when oedema was severe.

On one occasion only, a dog lung weighing 240 gms. was used as oxygenator for heart-lung by pass of a goat. This experiment has been excluded from the tables. The extreme ease with which this lung was ventilated and washed and the pure whiteness of its surface after washing compared with the ruminant lungs was most striking. There was a delay of three hours before this lung was used and although it oxygenated well, marked oedema was present after 15 minutes by pass at a flow rate of 1200 cc/min.

TABLE I

*Sheep's* lungs taken from abattoir and used within 2–3 hours in extra-corporeal circuit to perfuse *calf*.

| Expt.: No. | Pairs of lungs used | Donar heparin-ised | Pulmonary artery pressure of perfused lungs (max. reading) | Quality of wash with Dextran | Duration of per-fusion in minutes | Blood flow cc./min. | Oxygen-ation | Odema as per cent. weight increase | Fate of animal | Comment |
|---|---|---|---|---|---|---|---|---|---|---|
| 1 | | + | 60 | Good | 15 | 1,000 | Good | 80 | Died on table | Air embolus |
| 2 | 2 | + | 30 | Good | 15 | 1,000 | 100% | 37 | Died on table | Air embolus |
| 3 | 2 | + | 15 | Good | 25 | 1,000 | Good | 25 | Died during night | Pulmonary atalectasis |
| 4 | 2 | + | — | Fair | 50 | 1,000 | 95% | 42 | Died on table | Ventricular fibrillation |
| 5 | 2 | + | 45 | Good | 35 | 1,000 | 87% | 26 | Died 3 hours post. op. | Pulmonary atalectasis |
| 6 | 2 | — | 50 | Poor | 32 | 1,000 | Good | 60 | Died on table | Air embolus |
| 7 | 1 | — | 40 | Good | 42 | 1,000 | Good | 70 | Survived | |
| 8 | 2 | — | 70 | Good | 20 | 1,000 | 85% | ? | Died during night | Pulmonary atalectasis |
| 9 | 2 | — | 80 | Fair | 30 | 1,000 | 80% | 42 | Died during night | Pulmonary atalectasis |
| 10 | 1 | — | — | Good | 20 | 1,000 | Poor | 133 | Died on table | Gross haemorrhagic pulmonary oedema during bypass |

TABLE II

*Goat's* lungs taken in laboratory and used within $1\frac{1}{2}$ hours in extracorporeal circuit to perfuse *calf*. (No. 27, was a sheep.)

| Expt.: No. | Donor heparinised | Pulmonary artery pressure of perfused lung (max. reading) | Quality of Dextran wash | Duration of perfusion in minutes | Blood flow (max.) cc./min. | Oxygen per cent cent. sat$^{r}$. | Oedema as per cent weight increase | Fate of perfused animal | Cause of death of perfused animals |
|---|---|---|---|---|---|---|---|---|---|
| 11 | + | 70 | Good | 27 | 1,000 | 98 | None | Died 2 hrs. post-op. | Gross pulmonary oedema. |
| 12 | + | 45 | Good | 27 | 1,250 | | None | Died 4 hrs. post-op. | Pulmonary atalectasis |
| 13 | + | 50 | Good | 38 | 1,000 | 80 | 10% | Survived | |
| 14 | — | 55 | Good | 30 | 1,000 | 68 | None | Died 8 hrs. post-op. | Pulmonary atalectasis |
| 15 | + | 40 | Good | 37 | 1,000 | | 21% | Died 4 hrs. post-op. | Pulmonary atalectasis |
| 16 | + | — | Good | 37 | 620 | 100 | None | Died before wound closure | Gross pulmonary oedema |
| 17 | + | 60 | Good | 38 | 1,250 | | None | Survived | |
| 18 | + | 80 | Good | 52 | 1,000 | 96 | 29 | Died 6 hrs. post-op. | Pulmonary atalectasis |
| 19 | + | 70 | Good | 35 | 1,000 | 97 | 43 | Died before wound closure | Gross pulmonary oedema |
| 20 | + | — | Good | 50 | 500 | 94 | 112 | Died during night | Mod. atalectasis |
| 21 | + | 90 | Good | 26 | 1,000 | 92 | 36 | Died during night | Mod. atalectasis |
| 22 | + | 55 | Good | 30 | 1,000 | 98 | 47 | Survived | |
| 23 | + | 55 | Good | 36 | 1,000 | 92 | 100 | Died 1 hr. post-op. | Haemothorax |
| 24 | + | 95 | Good | 38 | 1,250 | 86 | None | Died during night | P.M. not recorded |
| 25 | + | — | Good | 41 | 1,000 | | 40 | Died during night | P.M. not recorded |
| 26 | + | — | Good | 40 | 1,000 | | 200 | Died before wound closure | Gross haemorrhagic pulmonary oedema |
| 27 | + | — | Good | 46 | 1,250 | 96 | 200 | Died at end of op. | Would not start breathing again |
| 28 | + | — | Good | 40 | 1,000 | | 140 | Died on table | Irreversible ventricular fibrillation |

*Microscopical section of lung taken from unheparinized sheep, perfused with dextran to wash out sheep's blood and then perfused with calf's blood for 42 minutes during the course of a heart-lung bypass—the calf survived.* (*Table I, No.* 7). *The Section shows marked interlobular oedema.*

*Microscopical section of lung taken from a calf which died about 6 hours after complete heart-lung bypass for 25 minutes, using a sheep lung as oxygenator. (Table I, No. 3). The section shows severe alveolar oedema with a coagulated albuminous exudate blocking the alveoli.*

*Acid base balance:* In nine experiments the pH of the blood was estimated at the end of perfusion: in six it was within the normal range (7·3–7·45), in one it was below normal (7·2) and in two it was above normal (7·47 and 7·53).

*Electrolytes:* In fifteen experiments in which proteins and electrolytes were estimated before during and after perfusion there were no significant changes in the sodium, chloride and protein levels. The potassium level was unchanged in eight, raised in five and lowered in two. In no cases were these changes of any magnitude.

*Haemolysis:* In twenty experiments the average reading for plasma Hb at the end of perfusion was 100 mg per cent.—the readings ranging from zero to 420 mgm per cent.

*Platelets:* In ten experiments in which the platelets were counted at the end of perfusion the counts were between 100,000 and 500,000 in seven, over 500,000 in two, and 70,000 in one. (The normal average for the calf ≡ 160,000).

*E.E.G. Recordings:* The electroencephalograph recordings excluded the presence of significant cerebral anoxia in all technically satisfactory experiments.

*Fall in body temperature during perfusion.* There was a fall in body temperature during by-pass averaging 4·4° C. Prior to use of the electric blanket the average temperature drop equalled 6°C. and after its introduction 3·5°C. The main cooling occurred as the blood passed through the lung; the lung was cooled during its washing with cold dextran and also by the oxygen with which it was inflated.

*Histological appearances.* Histological examination of the donor lungs after perfusion (Plate XXXIV) showed a variable degree of interlobular oedema and patchy collapse most noticeable in dependent parts of the lungs. The histological changes in the lungs of the calves dying after perfusion from apparent atelectasis (Plate XXXV) were of considerable interest. These lungs showed severe alveolar (not interstitial) oedema and haemorrhage, with fibrin plugs in the terminal bronchi in some places. The oedema fluid was highly albuminous and had coagulated, obstructing pulmonary ventilation very much in the same way as in hyaline membrane disease of the new born. The results of this was massive resorption collapse of the lungs and death. Similar changes however, were seen in the lungs of a calf dying after thoracotomy alone in which no perfusion had been done. (v.i.).

### DISCUSSION

When a heterologous lung is used as oxygenator in an extracorporeal circuit it is necessary to wash out the native blood before perfusing it with the blood of the operated subject. The lung of the Rhesus monkey appears to be an exception to this rule because its blood is compatible with human blood, and Mustard has used it successfully in this way without prior washing.

The technique of preparing the lung for use is complex, and many points must be observed in order to get a good result. The following lessons were learnt:

(1) The donor animal must be heparinized before killing as otherwise it is impossible to avoid some clotting in the lungs.

(2) The lungs must not be allowed to collapse during removal because they are difficult to reinflate and may be damaged in the attempt.

(3) The washing fluid must be of the same pH and osmotic pressure as blood. Dextran was found ideal for this purpose, isotonic saline and gum saline both produced oedema.

(4) The dextran should be pumped through the lung at a mean pressure not exceeding 20 mm. Hg., a relatively smooth flow seems to be better than a pulsatile flow.

(5) In making the cannulations air embolus must be scrupulously avoided.

(6) During washing the lungs should be rhythmically inflated, the pressure of gas must not exceed 20 cm. of water otherwise the alveolar membranes may be ruptured and pulmonary oedema will follow. (It was some time before we came to realize the importance of this point).

(7) Washing must be continued with repeated changes of dextran until the effluent is quite clear, about 15–20 pints are needed for the average goat lung. With care and attention to these details a lung almost completely free of blood and oedema was regularly obtained. It is worthy of mention that the dog lung seems to be easier to wash clean than that of the sheep or goat.

There should be as little delay as possible between the preparation and use of the lungs as an oxygenator. This requires careful timing and coordination. There is no doubt that the fresher the lung the longer it will stand up to perfusion without the development of oedema. Provided it is properly prepared and freshly used a goat lung can be relied upon to act efficiently as an oxygenator for 30 to 45 minutes with flows up to 1 litre per minute. Once oedema has begun to occur it develops fairly rapidly, though oxygenation may remain adequate for some time after the lungs have become obviously oedematous. Nevertheless oxygenation has been incomplete even when the lungs did not develop oedema to any extent. However, an oxygen saturation of 80 per cent. was found to be compatible with survival and in no case in which good flow was obtained did EEG evidence of cerebral anoxia develop. The results of analysis of pH, electrolyte balance and platelet survival lend support to the view that the heterologous lung produces no ill effect in an extra-corporeal circuit provided it is not used after it has become damaged by oedema.

On the debit side there are considerations of a practical nature which weigh heavily against the method. When applied to clinical use it is necessary to set up an animal operation theatre close to the main operating theatre for removal of the lungs under aseptic conditions. When the patient is an adult two animals must be sacrificed and two pairs of lungs taken. The introduction of two live calves or goats into hospital without causing undue comment presents problems! It takes 15 to 20 pints of dextran to wash out each pair of lungs, so that the cost in an adult case is about £30–£40 for dextran alone. The preparation of the machine for use takes several hours and it is not easy to co-ordinate with the surgeon so that both machine and surgeon are ready at the same time. The safe duration of the perfusion is from $\frac{1}{2}$–$\frac{3}{4}$ hour which, although long enough for most procedures, is not adequate for the more complicated cardiac anomalies that might come to surgery. Finally, during use the lungs become gradually oedematous and remove from the circulation an amount of fluid that cannot be estimated accurately until the lungs have been removed from the machine and weighed.

Considered in terms of survival these results have not been encouraging, for from twenty-five technically satisfactory experiments there were only four animals that survived completely. Lung complications were the commonest cause of death; five animals developed gross haemorrhagic pulmonary oedema either during or immediately after perfusion and died on the operating table (over-transfusion may have played a part in some of these but not in all). The remaining thirteen died some hours after operation, probably as a result of widespread resorption collapse of the lungs, due to a highly albuminous coagulated intra-alveolar exudate. No satisfactory explanation of these pulmonary deaths has been found. The possibility that it results from an anaphylactic reaction from foreign protein in the heterologous lung was rejected on the grounds that previous sensitisation was lacking. Furthermore a control experiment in which the operative part of the procedure was performed exactly as for perfusion but without any perfusion being carried out, (v.s.) showed that a major thoracotomy alone could be fatal in the calf, for this animal died within 12 hours and the lungs revealed the usual patchy collapse and familiar coagulated exudate within the alveoli. Ruminant lungs have a respiratory epithelium very prone to develop an exudative reaction. Perhaps the bovine species is particularly liable because pulmonary oedema occurs readily in cattle and can be due to a variety of causes, including mouldy forage, injection of foreign proteins, toxins and chemicals. Pulmonary oedema may also occur during massive infestation with the lung parasite *dictyocollis*. Cattle being shown at Smithfield Show during 1952 "Smog" died of pulmonary oedema while other species were unaffected. Our results are therefore difficult to assess and a success rate of 14 per cent. does not necessarily condemn the method. Perhaps it is surprising that the isolated ruminant lung functioned as well as it did as an oxygenator.

### SUMMARY

In twenty-eight experiments an isolated heterologous lung has been used as oxygenator in an extra-corporeal circuit.

Four animals survived out of twenty-three technically satisfactory experiments. Most of the deaths were due to pulmonary oedema to which ruminants seem to be unduly prone.

When carefully prepared, the heterologous lung has been shown to function well as an oxygenator. Practical considerations, however, weigh heavily against this method in human surgery.

### ACKNOWLEDGEMENTS

We wish to thank Professor C. W. Ottaway and Professor R. J. Brocklehurst who have made available to us laboratory facilities at the Veterinary School, University of Bristol, Professor A. Messervy for much helpful advice and assistance, Dr. O. C. Lloyd for examining histological sections, Dr. F. G. Bolton for haematological investigations, Dr. G. K. McGowan for biochemical investigations and Mr. Garry Meade who has made much of the apparatus for us.

Finally, we wish to thank the Nuffield Trust for financing this work, and Professor R. Milnes Walker who initiated and guided it.

### REFERENCES

Bjork, V. O. (1948). *Lancet*, **2**, 491.

Cleland, W. P., Melrose, D. G. (1955) Brit. Med. Bull. 11, 236.

Clutton-Brock, J. (1957). *Brit. J. Anaesth.* **29**, 517.

Denis, C., Spring, D. S., Nelson, G. E., Karlson, K. E., Nelson, R. M., Thomas, J. V., Eder, W. P., Varco, R. L. (1951). *Ann. Surg.* **134**, 709.

Dodrill, F. D., Hill, E., Gerisch, R. A. (1952). *J. Thor. Surg.*, **24**, 134.

Gibbon, J. H. Jr. (1953). *Recent Advances in Cardio-vascular physiology and surgery*, pp. 107–113. (Symposium presented by Minnesota Heart Association and University of Minnesota, Sept. 1953) Minneapolis, University of Minnesota, 1953.

Handforth, C. P., (1955) Lancet, 1, 1252.

Helmsworth, J. A., Clark, L. C., Sherman, R. T. Largen, L. (1952). *J. Thor. Surg.*, **24**, 117.

Jongbloed, J. (1949). *S.G.O.*, **89**, 684.

Melrose, D. G., (1953), *B.M.J.*, **2**, 57.

Mustard, W. T., Chute., Simmons, E. H. (1952). *Surgery*, **32**, 803.

Roughton, F. J. W., Scholander, P. F. (1943); J. Biol. Chem., **148**, 541.

## Figures and Tables

**Fig. 1. f1:**
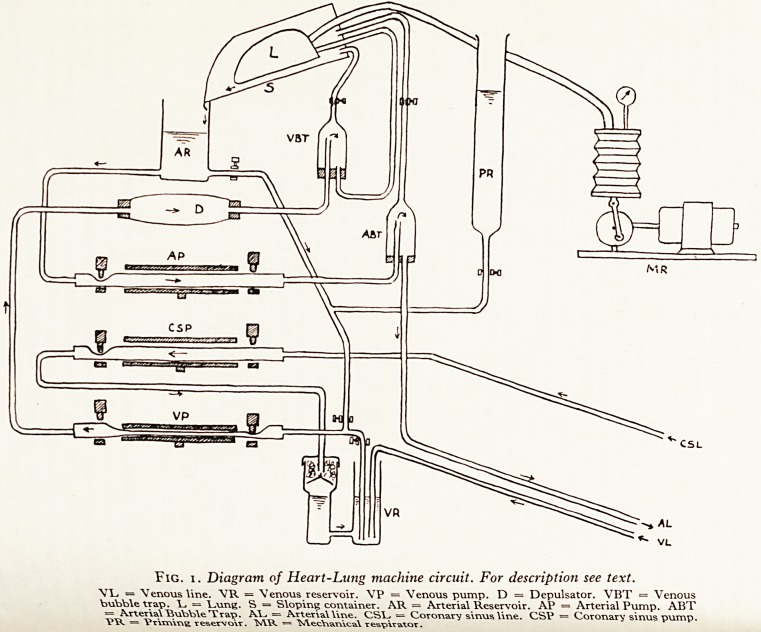


**Figure f2:**
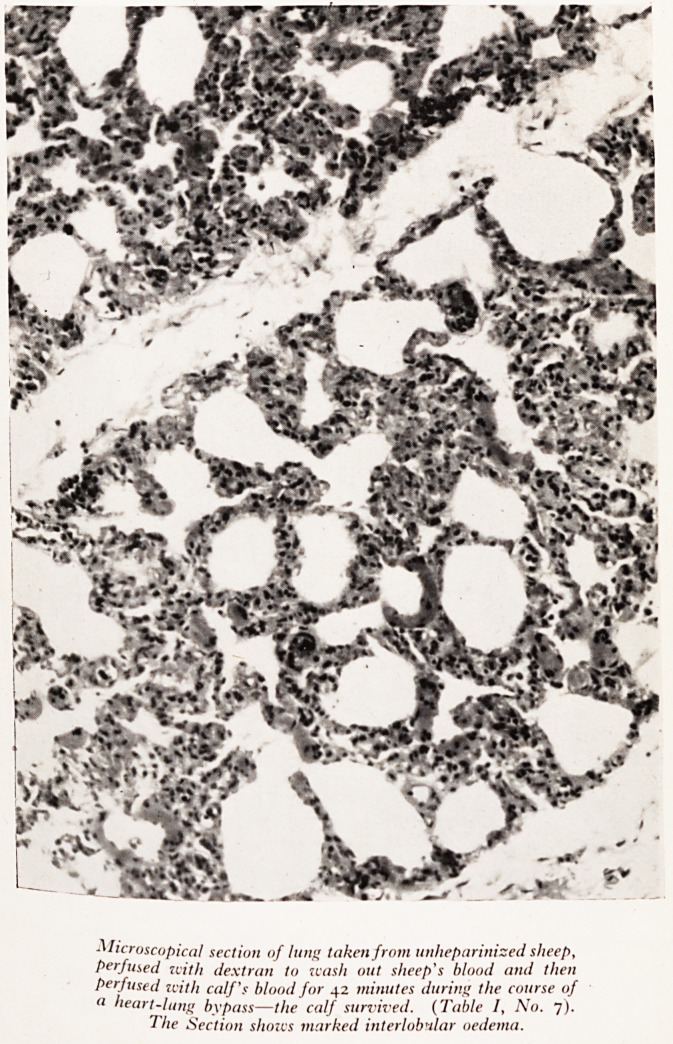


**Figure f3:**